# Is Zinc Accumulation Increased in Hyperplastic Compared to Normal Prostate Tissue

**DOI:** 10.3390/ijms27031466

**Published:** 2026-02-02

**Authors:** Tomislav Pejčić, Biljana Dojčinović, Milica Zeković, Uroš Bumbaširević, Tomislav Tosti, Živoslav Tešić, Lato Pezo, Darko Jovanović, Darko Laketić, Milica Kalaba

**Affiliations:** 1 Faculty of Medicine, University of Belgrade, dr Subotića 8, 11000 Belgrade, Serbia; urosbu@gmail.com (U.B.); drdarkolaketic@gmail.com (D.L.); 2 Clinic of Urology, University Clinical Center of Serbia, Pasterova 2, 11000 Belgrade, Serbia; darkojov@gmail.com; 3 Institute of Chemistry, Technology and Metallurgy, National Institute of the Republic of Serbia, University of Belgrade, Njegoševa 12, 11158 Belgrade, Serbia; bmatic@chem.bg.ac.rs (B.D.); tosti@chem.bg.ac.rs (T.T.); 4 Centre of Research Excellence in Nutrition and Metabolism, Institute for Medical Research, National Institute of Republic of Serbia, University of Belgrade, Tadeuša Košćuška 1, 11000 Belgrade, Serbia; zekovicmilica@gmail.com; 5 Faculty of Chemistry, University of Belgrade, Studentski trg 12-16, 11158 Belgrade, Serbia; ztesic@chem.bg.ac.rs; 6 Institute of General and Physical Chemistry, Studentski trg 12-16, 11158 Belgrade, Serbia; latopezo@yahoo.co.uk (L.P.); milicaffh@yahoo.com (M.K.)

**Keywords:** benign prostatic hyperplasia, zinc, testosterone, dihydrotestosterone, ICP-OES

## Abstract

In the male body, zinc accumulates most abundantly in prostatic cells, where it plays a key role in producing high amounts of citrate in seminal fluid. Intraprostatic accumulation of Zn increases during the development of benign prostatic hyperplasia (BPH), one of the most common diseases in men over 50 years of age. Continuing our investigations on intraprostatic androgens, in this study, we analyzed the mineral content (Zn, Ca, Cu, K, Mg, Mn, and Na) in the transitional zone (TZ) of the prostate using inductively coupled plasma optical emission spectrometry (ICP-OES). The concentrations of testosterone (T) and dihydrotestosterone (DHT) were determined by liquid chromatography–mass spectrometry (LC-MS). Group-wise and correlation analyses demonstrated a descriptive trend toward a volume-dependent increase in Zn concentrations within TZ tissue, whereas other elements exhibited heterogeneous covariance patterns; intraprostatic hormone levels, although elevated in larger prostates, showed no consistent linear correlations with elemental concentrations. Given the observational design of the present study, the reported tissue Zn profiles cannot be interpreted as evidence supporting supplementation in BPH, and any potential clinical implications warrant evaluation in rigorously designed interventional studies.

## 1. Introduction

In contemporary organisms, Zn is an essential trace element that underpins nearly all aspects of cellular function and is involved in the regulation of every phase of the cell cycle [1]. Zinc participates in multiple cellular signaling pathways and serves as an integral structural and catalytic component of numerous proteins, including transcription factors, growth factors, and receptors [2,3]. Approximately 3000 human proteins are known to bind Zn, in addition to several hundred proteins involved in its intracellular transport and signaling pathways [4,5,6]. Zinc is present in all major classes of enzymes and is essential for the catalytic activity of more than 300 enzymes and over 1000 transcription factors. Furthermore, zinc plays a key role in the regulation of apoptosis and is critically involved in RNA and DNA metabolism, as well as in gene expression [7].

In humans, this intricate homeostatic machinery is reflected in a specific tissue distribution pattern, with exceptionally high zinc concentrations characterizing seminal plasma, prostatic tissue, the eye, brain, muscles, bones, kidneys, and liver [8,9,10].

In human prostatic cells, Zn levels are tightly regulated by Zn transporters, ion channels, and Zn-sensing molecules [11,12]. Among these, the ZIP1 transporter is the principal mediator of zinc uptake under physiological conditions and plays a central role in maintaining the uniquely high zinc content of the prostate. Enhanced ZIP1 activity increases intracellular Zn accumulation, augments citrate production and exerts an inhibitory effect on cellular proliferation.

Zinc homeostasis in prostatic tissue is further shaped by androgenic and lactogenic stimuli: both testosterone and prolactin (PRL) stimulate the expression of zinc transporters and key enzymes within the citrate-producing metabolic pathway, thereby promoting Zn uptake and citrate synthesis [13,14,15,16]. Notably, zinc supplementation has been shown to enhance the 5α reduction of testosterone to dihydrotestosterone, underscoring the bidirectional interplay between zinc metabolism and androgen action [17].

Zinc accumulates in prostatic secretory epithelial cells, primarily within the mitochondria, where it is present at concentrations up to 20-fold higher than in other cell types. Citrate synthesis from aspartate and glutamate also occurs in the mitochondria of these secretory cells. The massive production of citrate is directly dependent on high intracellular Zn levels [18,19]. Acting as a metabolic gatekeeper, Zn inhibits mitochondrial aconitase and thereby halts the progression of citrate through the Krebs cycle, ensuring its retention and remarkable intracellular accumulation in prostatic secretory epithelium [14]. Consequently, citrate is present in the prostate at concentrations approximately 100-fold higher than in other soft tissues.

**Figure 1:**
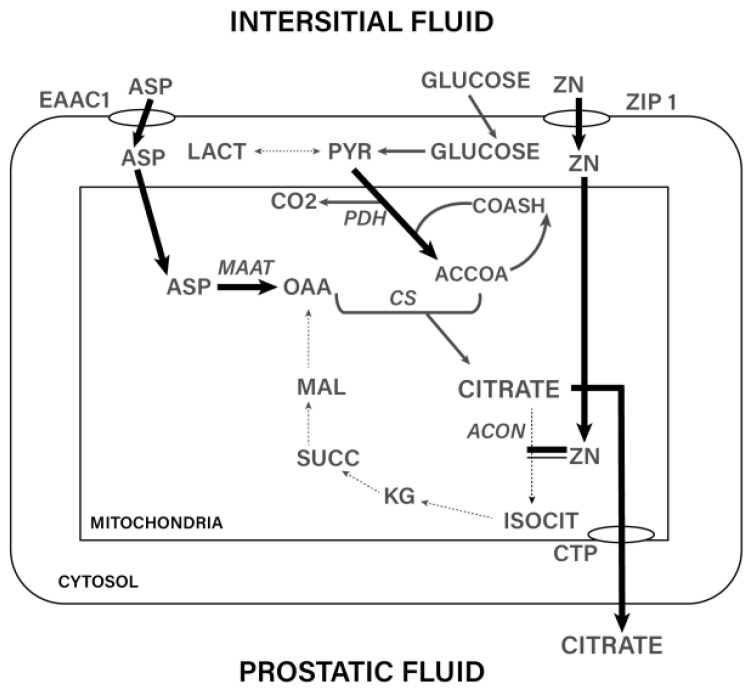
Schematic representation of zinc uptake, mitochondrial compartmentalization, and citrate-centered metabolic organization underlying the distinctive secretory phenotype of human prostatic epithelial cells. Zinc is imported predominantly via ZIP1 and accumulates within mitochondria, where inhibition of mitochondrial aconitase limits citrate oxidation and functionally truncates the tricarboxylic acid cycle. Aspartate-derived oxaloacetate and glycolytically generated acetyl-CoA are condensed by citrate synthase to form citrate, which accumulates intracellularly and is subsequently exported into prostatic fluid via the citrate transport protein. Downstream metabolic intermediates of the tricarboxylic acid cycle are depicted for contextual completeness but remain functionally suppressed in the prostate due to zinc-mediated aconitase inhibition. Abbreviations: LACT, lactate, CO_2_, carbon dioxide; ACCOA, acetyl-CoA; ACON, aconitase; ASP, aspartate; CTP, citrate transport protein; CS, citrate synthase; EAAC1, excitatory amino acid carrier 1; ISOCIT, isocitrate; KG, α ketoglutarate; SUCC, succinate; MAL, malate; MAAT, mitochondrial aspartate aminotransferase; ACON, mitochondrial aconitase; OAA, oxaloacetate; PDH, pyruvate dehydrogenase; PYR, pyruvate; SUCC, succinate; ZN, zinc; ZIP 1, Zrt-/Irt-like protein 1; COASH, coenzyme A (free thiol form).

The schematic (Figure 1) depicts the coordinated metabolic and transport processes that underlie the unique citrate-producing phenotype of prostatic secretory epithelial cells. Zinc is taken up from the interstitial fluid predominantly via the ZIP1 transporter and accumulates within mitochondria, where high Zn concentrations inhibit mitochondrial aconitase (m-aconitase). This Zn-dependent blockade truncates the Krebs cycle at the citrate → isocitrate step, preventing citrate oxidation and enforcing marked intracellular citrate accumulation. Citrate is subsequently exported to the prostatic fluid through the citrate transport protein (CTP). Aspartate (ASP), imported through the EAAC1 transporter, is converted to oxaloacetate (OAA) via mitochondrial aspartate aminotransferase (mAAT), while glucose metabolism supplies pyruvate and acetyl-CoA through glycolysis and pyruvate dehydrogenase (PDH). Citrate synthase (CS) condenses OAA and acetyl-CoA to generate citrate. Although downstream Krebs cycle intermediates (isocitrate, α-ketoglutarate, succinate, malate) are shown for contextual completeness, they do not participate meaningfully in citrate oxidation in the prostate due to Zn-mediated inhibition of aconitase. The resulting high intracellular citrate pool is characteristic of the normal and hyperplastic prostate and underpins its physiological secretion into seminal fluid (Figure 1).

In seminal plasma, Zn concentrations range from 50 to 250 µg/mL, while in normal prostatic fluid they reach 350–500 µg/mL; in prostatitis, Zn levels decrease to approximately 50 µg/mL [20,21]. Zinc is secreted from the prostate primarily as a complex with citrate. Following ejaculation, approximately 50% of Zn is redistributed and binds to various compounds originating from the seminal vesicles [22].

Zinc exerts antibacterial effects in human semen and regulates the activity of numerous proteins and enzymes, including prostate-specific antigen (PSA) [23,24]. Seminal citrate concentrations are markedly higher in healthy men (≈15.5 mM) compared with patients with prostate cancer (≈3.9 mM) [25]. Within the seminal milieu, citrate plays a dual regulatory role: it contributes to buffering the pH and modulates calcium levels. Calcium, in turn, is essential for flagellar function and progressive sperm motility, while the maintenance of a near-neutral pH ensures that sperm remain motile and functionally competent despite the inherently acidic environment of the vagina [26,27].

In benign prostatic hyperplasia, altered zinc homeostasis has been reported, characterized by increased serum zinc concentrations, suggesting dysregulation of zinc metabolism rather than unequivocal intraprostatic accumulation [28]. In the prostate, zinc accumulation occurs predominantly in epithelial cells and, to a lesser extent, in fibroblasts [29,30,31]. However, Zn levels are significantly reduced in prostate cancer (PCa) tissue due to impaired cellular metabolism [32]. In malignant cells, low expression of ZIP1 is associated with decreased Zn accumulation and enhanced citrate oxidation [33]. Numerous studies have demonstrated that Zn levels in PCa are reduced by 70–90% compared with normal prostate tissue [34,35,36]. Consequently, Zn deficiency in PCa is considered more likely a consequence of malignant transformation rather than its primary cause [33,37].

Although numerous studies have explored Zn concentrations in normal, hyperplastic, and malignant prostate tissue, comparability across reports is limited due to heterogeneous sampling strategies, differing analytical platforms, and the frequent use of autopsy or surgical material rather than fresh biopsy tissue. Notably, only a few investigations have quantified zinc and other essential metals specifically within the transition zone, despite its central role in BPH pathophysiology.

Accordingly, we sought to define the intratissue concentrations of zinc, selected macro- and microelements, and key intraprostatic androgens in transition-zone biopsy specimens obtained during routine transrectal ultrasound (TRUS)-guided prostate biopsy. We hypothesized that zinc accumulation within the transition zone would increase proportionally with prostate volume and mirror local testosterone and dihydrotestosterone levels, whereas metals not governed by androgen-dependent regulatory pathways would exhibit no appreciable variation.

## 2. Results

### 2.1. Data Analysis for Different Prostate Volume

Among the 82 patients included in the study, 36 had a small prostate (total prostate volume, TPV ≤ 30 mL), while 46 had an enlarged prostate (TPV > 30 mL) [38]. The results for all patients are presented in Table S1, while the descriptive analysis is shown in Table 1.

Pathohistological analysis revealed prostate cancer in 42 patients, while 39 patients showed no evidence of PCa. In patients with small prostates, the median Zn concentration was lower than in those with enlarged glands (73.19 µg/g vs. 79.70 µg/g, respectively, Table 1). Notably, Zn levels did not differ between patients with and without PCa, consistent with the predominantly peripheral localization of malignancy and systematic sampling from non-malignant TZ regions. Tissue concentrations of other metals did not differ significantly between small and large prostates. Calcium concentrations were slightly higher in large prostates compared with small prostates. The concentrations are presented in Table 1. In contrast, the median concentrations of T and DHT in the TZ of enlarged prostates were higher (0.79 ng/g and 14.45 ng/g, respectively) than those in small prostates (0.44 ng/g and 4.74 ng/g, respectively), Table 1.

Both Shapiro–Wilk and Anderson–Darling tests indicated significant deviations from normality for all analyzed variables (*p* < 0.05). Consequently, parametric assumptions were not met, and non-parametric statistical methods were considered appropriate. However, group comparison tests could not be performed due to the absence of a valid grouping structure in the analyzed dataset.

Due to non-normal data distribution, differences between TPV groups were assessed using the Mann–Whitney U test. Statistically significant differences were observed for DHT (*p* < 0.001) and T (*p* < 0.001), with large (r = 0.699) and moderate to large (r = 0.485) effect sizes, respectively. In contrast, no significant differences were detected for Ca, K, Mg, Na, Cu, Mn, or Zn (*p* > 0.05), and their effect sizes were negligible to small.

### 2.2. Data Analysis for the Entire Cohort

The descriptive statistical analysis of elemental and hormonal concentrations in transition-zone prostate tissue (*n* = 82) revealed marked inter-individual variability across all examined parameters.

Zinc (Zn) concentrations showed a median value of 73.23 µg/g wet weight (w.w.), with values ranging from 15.61 to 219.62 µg/g, indicating substantial dispersion across the cohort. Median Zn levels were comparable between small and enlarged prostates, despite wider variability observed in the latter.

Calcium (Ca) exhibited the highest tissue concentrations, with a median value of 590.18 µg/g and a broad range from 156.53 to 5814.15 µg/g, reflecting pronounced heterogeneity among patients. Major electrolytes demonstrated similarly wide distributions. Sodium (Na) concentrations had a median of 660.10 µg/g, ranging from 220.09 to 7239.93 µg/g. Potassium (K) and magnesium (Mg) displayed somewhat narrower, yet still substantial, variability, with median values of 451.35 µg/g (range: 151.04–1678.56 µg/g) and 63.62 µg/g (range: 11.06–201.23 µg/g), respectively.

Among trace elements, copper (Cu) and manganese (Mn) were present at lower absolute concentrations but exhibited notable relative variability. Copper showed a median concentration of 1.56 µg/g (range: 0.43–11.93 µg/g), while manganese had a median value of 0.70 µg/g, with concentrations ranging from 0.26 to 3.33 µg/g.

Hormonal markers also demonstrated substantial dispersion. Testosterone concentrations had a median value of 0.83 ng/g, ranging from 0.13 to 3.12 ng/g. Dihydrotestosterone (DHT) exhibited higher tissue levels, with a median concentration of 10.20 ng/g and a wide range from 0.99 to 24.95 ng/g.

Given the non-normal distribution and pronounced inter-individual variability of elemental and hormonal concentrations, median values and ranges were used to summarize the data.

Considering the substantial heterogeneity of elemental and hormonal distributions, subsequent analyses evaluated both differences between prostate volume groups and correlations among tissue elemental concentrations, androgen levels, and total prostate volume.

The corresponding variance values confirm these wide distributions, particularly for Ca (var = 990,994.36), Na (var = 984,937.33), and Zn (var = 1881.21).

### 2.3. Correlation Analysis

**Figure 2:**
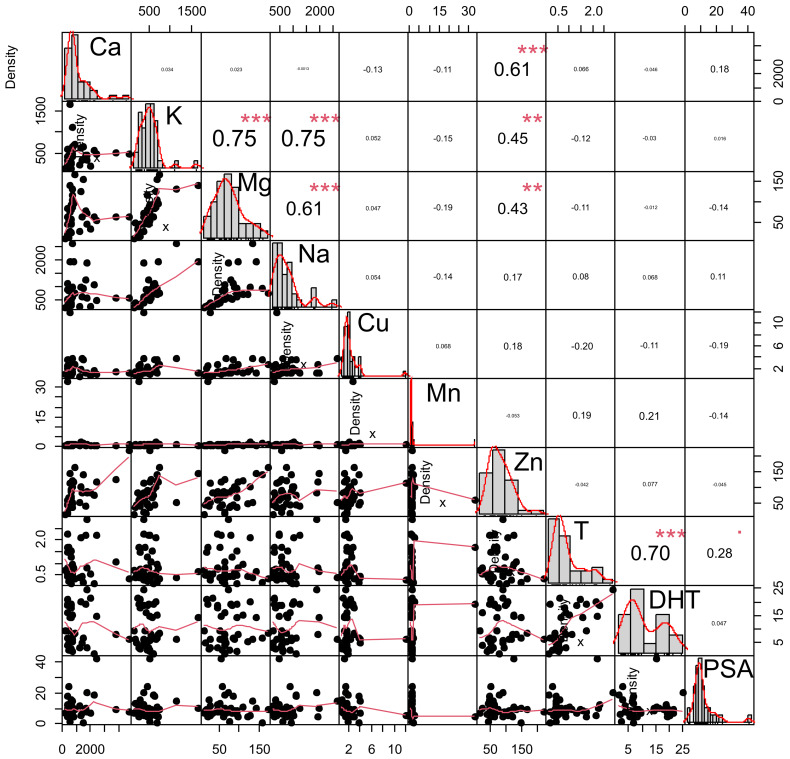
Correlation matrix of elemental and hormonal concentrations in prostatic transition-zone tissue (*n* = 82); Asterixs-statistically significant at *p* < 0.01 level (**), *p* < 0.001 level (***).

We next applied correlation analysis to delineate patterns of covariance among tissue elements and to explore potential associations between intraprostatic androgens and elemental concentrations across the full cohort. Within the correlation framework, Zn levels showed significant associations with several other elements, indicating that Zn accumulation occurs within a broader pattern of mineral variability rather than as an isolated phenomenon. The detailed correlation analysis provided a comprehensive numerical overview of these inter-element relationships (Figure 2).

Calcium exhibited a strong positive correlation with Zn (r = 0.619, *p* < 0.001), indicating parallel accumulation of both elements in hyperplastic tissue. Calcium also correlated significantly with Mg (r = 0.297, *p* = 0.007) and Mn (r = 0.225, *p* = 0.042), suggesting linked metabolic pathways in mineral regulation.

Potassium showed very strong associations with Mg (r = 0.772, *p* < 0.001) and a moderately strong association with Na (r = 0.475, *p* < 0.001), consistent with the physiological co-dependence of these electrolytes.

Magnesium further correlated with Na (r = 0.481, *p* < 0.001) and presented a notable positive correlation with Mn (r = 0.343, *p* = 0.002).

Copper demonstrated a moderate positive correlation with Zn (r = 0.244, *p* = 0.027) and a borderline association with Mn (r = 0.210, *p* = 0.058), indicating partial co-regulation among trace elements.

Zinc was significantly correlated with Mg (r = 0.394, *p* < 0.001), Mn (r = 0.303, *p* = 0.006), and Ca, suggesting that Zn variability within TZ tissue is embedded within a broader pattern of inter-element associations rather than representing an isolated phenomenon.

In contrast, hormone-related parameters exhibited minimal associations with element concentrations. Testosterone showed no significant correlations with any mineral, while DHT correlated solely with testosterone (r = 0.604, *p* < 0.001), indicating that hormonal status is largely independent of the elemental composition of prostate tissue.

## 3. Discussion

Inter-study comparisons of zinc concentrations in human prostatic tissue remain inherently challenging due to substantial methodological heterogeneity. Reported values vary not only because some authors quantify Zn on a dry-weight basis while others use wet-weight tissue, but also due to differences in sampling strategy (biopsy vs. transurethral resection of the prostate (TURP) vs. autopsy material), anatomical localization of the specimen, and analytical platforms ranging from atomic absorption spectrophotometry to energy-dispersive X-ray fluorescence (EDXRF) and modern ICP-based techniques. These factors complicate direct numerical comparison and likely account for part of the wide dispersion in published Zn values. A summary of literature-reported Zn concentrations in normal prostate tissue, BPH, and PCa is provided in Table 2. 

Early investigations suggested relatively modest Zn concentrations in normal prostate tissue (0.5–0.9 µg/g dry weight), with notably higher levels in secretory epithelium compared with stromal components [39]. Subsequent studies, most prominently those by Zaichick and colleagues [36], demonstrated high Zn content in BPH, typically exceeding 1100 µg/g dry weight, whereas malignant tissue consistently exhibited markedly reduced concentrations (≈146 µg/g dry weight). Autopsy studies further extended this observation, reporting Zn concentrations above ~1000 µg/g dry weight in histologically normal glands [40,41].

**Table 2:** Literature summary of zinc concentrations in normal prostate tissue, benign prostatic hyperplasia (BPH), and prostate cancer (PCa), expressed on a wet weight and dry weight basis. Data are reported as provided in the original references: single values may represent means or medians, while ranges indicate minimum–maximum values.

Reference	Zinc Tissue Concentration (µg/g Wet Weight)		
	Normal Tissue	BPH	PCa
[42]	211 (dorsal) (Autopsy)		
	184 (lateral) (Autopsy)		
	110 (medial) (Autopsy)		
[43,44]	30–35 (young) (Autopsy)		
	189 (31–50 years) (Autopsy)		
	220 (51–70 years) (Autopsy)		
[19,35]	196–294 (PZ)		26.2–52.3
	121 (CZ)		
[45]	100 (Autopsy)	200 (TURP)	65 (TURP)
[46]	215 (Autopsy)	105 (TURP)	60 (RRP)
[47]		77.9 (TURP)	49.2 (RRP)
[48]	84.6–240 (Autopsy)	350.6 (Autopsy)	28.7 (Autopsy)
[49]	109 (Biopsy)		
[40,41]	1018 (Autopsy)	1142–1235 (Biopsy)	146 (Biopsy)
Current research	73.19 (Biopsy, TZ)	79.70 (Biopsy, TZ)	

TZ—transitional zone; CZ—central zone; TURP (transurethral resection of the prostate); RRP (radical retropubic prostatectomy).

Age-dependent anatomical variation has also been described. In autopsy material, Tisell et al. [42] found that Zn concentrations were significantly higher in the dorsal (211 µg/g w.w.) and lateral lobes (184 µg/g w.w.) in individuals aged 51–70 years compared with the medial lobe, where concentrations were 110 µg/g w.w. Zaichick et al. [43] also reported an age-related increase in Zn levels: 30–35 µg/g w.w. in young men, 189 µg/g w.w. in men aged 31–50 years, and 220 µg/g w.w. in men aged 51–70 years (Table 2). The analytical method used in these studies was energy-dispersive X-ray fluorescence a method that quantifies total elemental content but is influenced by tissue density, fixation, and post-mortem alterations, thereby limiting comparability with fresh biopsy-based measurements [43,44].

A substantial body of literature underscores that zinc distribution within the prostate is highly zone-dependent and profoundly altered by benign and malignant pathology. Costello and colleagues [19,35] demonstrated that the peripheral zone (PZ), which contains the highest density of fully differentiated secretory epithelium, exhibits the greatest Zn content, typically ranging from 196 to 294 µg/g wet weight, whereas the central zone (CZ) contains intermediate levels (~121 µg/g). In stark contrast, malignant tissue from the PZ displays a precipitous decline in Zn concentration (26–52 µg/g w.w.) (Table 2), reflecting the well-established metabolic reprogramming accompanying tumorigenesis. The extraordinarily high Zn content of prostatic fluid (≈589 µg/mL) further attests to the physiological specialization of secretory epithelial cells in the healthy gland.

Findings from autopsy and surgical series reinforce this zonal and pathological gradient. Sapota et al. [45] reported approximately 100 µg/g w.w. in normal prostate tissue, rising to ~200 µg/g in BPH tissue and falling to ~65 µg/g in cancer obtained during TURP.

Christudoss et al. [46] observed comparable patterns: autopsy-derived normal prostate contained ~215 µg/g w.w., BPH tissue ~105 µg/g, and malignant tissue after radical prostatectomy ~60 µg/g (Table 2). Similar trends were confirmed by Singh et al. [47], who documented significantly higher Zn levels in BPH than in prostate cancer (77.9 ± 43.4 vs. 49.2 ± 29.3 µg/g w.w., Table 2, respectively), using atomic absorption spectrophotometry. Although absolute values varied across studies, the directionality was consistent: BPH exhibits preserved or elevated Zn accumulation, whereas prostate cancer is marked by profound Zn depletion—a biological signature linked to ZIP1 downregulation and restored citrate oxidation in malignant cells.

In a 2021 autopsy study, Daragó et al. [48] reported that zinc concentrations in men younger than 35 years were 84.6 µg/g wet tissue in the central zone and 114.4 µg/g wet tissue in the peripheral zone. In men aged ≥36 years, zinc concentrations increased to 240 µg/g wet tissue in the central zone and 117.2 µg/g wet tissue in the peripheral zone. In patients with BPH, Zn concentrations in the central zone reached 350.6 µg/g wet tissue, while in the peripheral zone they were 87.9 µg/g wet tissue. In men with histologically confirmed prostate cancer, Zn concentrations were 231.4 µg/g wet tissue in the central zone and only 28.7 µg/g wet tissue in the peripheral zone. These findings (also summarized in Table 2) demonstrate an age-related accumulation of Zn, with the highest concentrations observed in BPH tissue. Consistent with previous studies, Zn concentrations were lowest in the peripheral zone of the prostate, corresponding to malignant tissue [48].

In a 2021 systematic review of 105 studies, Zaichick [49] reported that zinc concentrations in normal prostate glands ranged from 17 to 547 µg/g, with a median of 109 µg/g on a wet weight basis (Table 2).

Taken together, Zn concentrations in normal prostate tissue from autopsy material range from 100 to 220 µg/g w.w. and tend to increase with age. In the transition zone, Zn concentrations range from 110 to 121 µg/g w.w., whereas in the peripheral zone, they are higher, ranging from 196 to 294 µg/g w.w. In BPH tissue, Zn concentrations are the highest, ranging from 105 to 589 µg/g w.w., while the lowest concentrations are found in PCa tissue, ranging from 26 to 65 µg/g w.w.

Against this heterogeneous backdrop, our methodological approach offers several advantages. Tissue was obtained exclusively from the TZ under real-time TRUS guidance, thereby minimizing inadvertent contamination from PZ tissue, which is both metabolically distinct and more likely to harbor occult malignancy. Immediate snap-freezing and storage at –70 °C preserved the biochemical integrity of the samples, and quantification by ICP-OES provided high analytical precision. Expressing Zn concentrations strictly on a wet-weight basis further enhances comparability with contemporary biopsy-based studies.

In our study, the median zinc concentration in biopsy-derived transition-zone tissue was 73.23 µg/g wet weight. This value falls within the range reported in previous biopsy- and surgery-based investigations [47], despite differences in tissue source and potential electrothermal effects associated with surgical procedures such as TURP. Moreover, our findings are broadly consistent with values summarized in recent systematic reviews of prostate zinc content [49]. Nevertheless, direct numerical comparisons with studies based on autopsy material remain limited due to post-mortem tissue dehydration and related compositional changes, as well as methodological differences in zinc expression (wet vs. dry weight). The absence of linear correlations between intraprostatic androgen concentrations and elemental levels does not preclude biological interaction, but may reflect the limited sample size, inter-individual heterogeneity, and the complexity of androgen-mediated regulatory mechanisms at the tissue level.

Our findings highlight several methodological and biological insights. First, TRUS-guided prostatic biopsy provides a reliable means of obtaining fresh, anatomically well-defined prostatic tissue, thereby avoiding many of the post-mortem and zonal confounders present in earlier studies. Within this precisely sampled TZ tissue, we observed a clear volume-dependent increase in zinc concentration. This gradient is fully aligned with the established androgen-regulated metabolic phenotype of secretory epithelial cells, in which higher intraprostatic testosterone and dihydrotestosterone concentrations promote zinc uptake, mitochondrial accumulation, and the suppression of citrate oxidation.

Importantly, this androgen-linked behavior was specific to zinc. Concentrations of metals whose cellular handling is not governed by androgen signaling remained stable across prostate sizes, underscoring zinc’s unique position within the prostatic metabolic network. The only exception was calcium, which showed a modest upward trend in larger prostates—an observation consistent with its partial co-regulation by androgen-driven pathways.

Collectively, the data indicate that prostate tissue exhibits high inter-individual variability in elemental composition, with zinc concentrations demonstrating considerable spread but remaining consistently elevated across the cohort, supporting the interpretation that zinc accumulation is substantially increased in hyperplastic compared with normal prostate tissue. To obtain additional insight, correlation analysis was performed on the entire data set. Taken together, these findings emphasize that prostate enlargement is accompanied not only by increased zinc accumulation but also by more subtle, coordinated shifts in tissue mineral composition, underscoring the metabolic complexity of benign prostatic hyperplasia.

## 4. Materials and Methods

### 4.1. Ethical Considerations for Study Participants

This study was conducted in strict compliance with the ethical principles outlined in the Declaration of Helsinki and in accordance with applicable national regulations governing biomedical research involving human participants. Prior to inclusion, all participants received comprehensive information regarding the study objectives, procedures, and potential risks, and provided written informed consent. The study protocol was reviewed and approved by the Ethics Committee of the University Clinical Center of Serbia, Belgarde, Serbia (No. 1880/84, dated 25 December 2025), with the consent of the Professional Board of the Clinic of Urology of the University Clinical Center of Serbia, Belgarde, Serbia (No. 1033, dated 24 December 2025).

### 4.2. Study Population and Sample Collection

In this single-center study, intraprostatic concentrations of metals (Zn, Ca, Cu, K, Mg, Mn, and Na), as well as testosterone and dihydrotestosterone, were prospectively determined in 82 patients scheduled for initial prostate biopsy.

Exclusion criteria were defined to eliminate conditions capable of altering intraprostatic trace-element homeostasis or androgen metabolism. Patients with acute prostatitis or urinary tract infection, chronic pelvic pain syndrome, or any active inflammatory condition of the genitourinary tract were excluded due to their known impact on zinc dynamics. Individuals receiving androgen deprivation therapy, antiandrogens, exogenous testosterone, or 5α-reductase inhibitors were also excluded, as were those with recent (≤3 months) supplementation with Zn, Mg, Ca, Se, or multivitamin preparations that could confound tissue metal measurements. Additional exclusions comprised a history of prostate surgery or ablative therapy, chronic kidney or liver disease, malabsorptive disorders, and any systemic condition or malignancy likely to perturb steroid hormone concentrations or trace-element distribution. Participants with incomplete clinical, laboratory, or histopathological data required for predefined analyses were also excluded.

The indications for biopsy were abnormal digital rectal examination and/or prostate-specific antigen levels > 4 ng/mL, with no prior 5α-reductase inhibitor (5α-RI) therapy. Total prostate volume was determined by transrectal ultrasound using standard ellipsoid measurements. Prostates were categorized as small or enlarged using a TPV threshold of 30 mL, a convention widely adopted in contemporary urological practice to distinguish physiologic from hyperplastic gland size. In all analyses, a “small prostate” was defined as TPV ≤ 30 mL and a “large prostate” as TPV > 30 mL; this categorization was applied consistently across text and tables [38].

During TRUS-guided prostate biopsy of the peripheral zone, two additional samples from the transition zone were obtained for the quantitative determination of metal and androgen concentrations. All biopsies were performed by four experienced urologists following an identical standardized protocol to minimize inter-operator variability. TZ sampling was guided by predefined anatomical landmarks on axial and sagittal planes, ensuring consistent localization across patients. Care was taken to obtain samples exclusively from the TZ and to avoid the PZ due to the possible presence of prostate cancer. The TZ tissue specimens were removed from the biopsy needle immediately after collection, transferred into pre-weighed 1.5 mL Eppendorf tubes, snap-frozen in liquid nitrogen, and stored at −70 °C until further analysis. Wet weight was used for all quantitative determinations.

### 4.3. Reagents

Nitric acid (HNO_3_, 65 wt.%, Suprapur^®^) was purchased from Merck KGaA (Darmstadt, Germany). Ultrapure water (conductivity 0.05 µS/cm) was produced using a Barnstead™ GenPure™ Pro system (Thermo Scientific, Waltham, MA, USA).

Testosterone (99.9%), trideuterated testosterone D 3 (isotopic purity 98 atom% D), dihydrotestosterone and trideuterated dihydrotestosterone D 3 (isotopic purity 98 atom% D) were from Sigma-Aldrich (Steinheim, Germany). Methanol, ethyl acetate, acetonitrile, and formic acid were obtained from Merck (Darmstadt, Germany). ESI-L-Low Mix Tunning Concentration (G-1969 85000), API Reference Concentration (G-1969 85001), and Zorbax Eclipse Plus C18 (100 × 2.1 mm i.d.; 1.8 μm) were from Agilent Technologies (Waldbronn, Germany).

### 4.4. Determination of Zinc and Other Macro- and Microelements in Prostatic Tissue

Digestion of prostate tissue samples was performed using an advanced microwave digestion system (ETHOS 1, Milestone, Italy) equipped with an HPR-1000/10S high-pressure segmented rotor. Samples were processed in pressure-resistant polytetrafluoroethylene (PTFE) vessels (100 mL) fitted with QS-50 quartz inserts to ensure chemical inertness and minimal trace-metal contamination.

Following cryopreservation, samples were maintained continuously in a frozen state and digested without any intermediate thawing. All measurements were completed within six months of collection. To prevent trace-metal contamination, only metal-free polypropylene consumables were used. No metallic instruments came into contact with tissue after needle extraction. Time from tissue retrieval to cryopreservation in liquid nitrogen was consistently <10 s.

Approximately 10–50 mg of each prostate tissue sample was precisely weighed (accuracy ± 0.1 mg), placed into quartz inserts, and mixed with 5 mL of nitric acid. The temperature was gradually increased to 180 °C over the first 10 min using microwave irradiation, maintained at 180 °C for the subsequent 20 min, and then rapidly cooled to room temperature.

After cooling and without filtration, the digested solution was diluted to a final volume of 10 mL in the same quartz insert using ultrapure water.

### 4.5. ICP-OES Measurements

The concentrations of Zn and other macro- and microelements in the digested solutions were determined using ICP-OES (iCAP 6500 Duo, Thermo Fisher Scientific, Cambridge, UK). External calibration was performed using certified: Multi-element plasma standard solutions 4 (Specpure^®^, 1000 µg/mL; Alfa Aesar GmbH & Co. KG, Karlsruhe, Germany). The instrumental operating conditions for ICP-OES are summarized in Table S3. Each digested sample was measured in triplicate (*n* = 3). Measurement reliability was confirmed by a relative standard deviation below 0.5%. The limits of detection (LOD) and quantification (LOQ) were calculated based on calibration curve statistics (3σ and 10σ criteria, respectively) and are provided in the Supplementary Material (Table S4), expressed both in µg/L for the solution and recalculated to µg/g w.w. for prostate tissue. Analytical quality control (QC) was ensured using certified reference material (CRM) for trace metals in fish protein (DORM-4, National Research Council Canada, Ottawa, ON, Canada) selected for its matrix similarity to the analyzed biological samples (prostate tissue). Recoveries of measured concentrations relative to certified values ranged from 97% to 102% (Table S5). Final metal concentrations in prostate tissue samples were expressed in µg/g (w.w.), calculated based on the sample preparation protocol.

### 4.6. Determination of Testosterone and Dihydrotestosterone in Prostatic Tissue

The LC system was coupled to an Agilent 6120 Time-of-Flight mass spectrometer. Data acquisition and processing were carried out using MassHunter Workstation software, version B.07.00 (Agilent Technologies, CA, USA). All instrumental parameters, chromatographic conditions, and validation procedures were identical to those reported in the original publication [50].

### 4.7. Statistical Analysis

Normality of data distribution was evaluated using the Shapiro–Wilk and Anderson–Darling tests within TPV groups. Since all variables significantly deviated from normal distribution (*p* < 0.05), non-parametric statistical methods were applied. Results are presented as median and interquartile range (Q1–Q3). Differences between TPV groups were assessed using the Mann–Whitney U test. Statistical analyses were performed in R software, version 4.4.3, with the level of significance set at *p* < 0.05. The detailed correlation analysis on inter-elemen.t relationships and hormonal variables (Zn, Ca, Cu, K, Mg, Mn, Na, T, and DHT) was performed in R 4.4.3, using the Performance Analytics package (chart.Correlation).

## 5. Conclusions

In our previous study, we demonstrated that testosterone and dihydrotestosterone (DHT) are present at higher concentrations in hyperplastic stroma compared with normal prostatic stroma. Based on these findings, our working hypothesis was that zinc (Zn) accumulation would also be higher in hyperplastic than in normal stroma due to the androgen-rich microenvironment. However, our results showed no statistically significant differences in Zn concentrations between small and large prostates, that is, between normal and hyperplastic stroma.

Moreover, the observed correlations between Zn and other metals, particularly calcium (Ca), suggest that metal accumulation in prostatic tissue reflects distinct metabolic processes that continue to occur in hyperplastic prostate tissue. Consequently, our initial assumption that Zn supplementation—considered beneficial in prostate cancer and chronic prostatitis—may not be necessary in benign prostatic hyperplasia does not appear to be strongly supported by our findings.

Nevertheless, further studies are warranted to elucidate the relationship between Zn concentrations in chronic prostatitis and prostate cancer, to determine whether these alterations represent a cause or a consequence of disease, and to assess whether Zn supplementation may offer potential therapeutic benefit.

To the best of our knowledge, this study is the first to compare Zn and other metal concentrations in normal and hyperplastic prostatic tissue using fresh samples from the transitional zone. We intend to extend this research by analyzing surgical specimens from both the transitional and peripheral zones of patients with benign prostatic hyperplasia and prostate cancer.

## Figures and Tables

**Table 1:** The descriptive statistical analysis of seven elements (µg/g), testosterone (T, ng/g), dihydrotestosterone (DHT, ng/g), measured in the transition zone tissue of small (TPV ≤ 30 mL) and large (TPV > 30 mL) prostates and prostate-specific antigen (PSA, ng/mL) in serum.

Parameter	Ca	K	Mg	Na	Cu	Mn	Zn	T	DHT	PSA
TPV ≤ 30 mL										
Median	659.26	504.17	70.31	660.10	1.77	0.93	73.19	0.44	4.74	9.20
Min	156.53	220.11	13.64	221.72	0.43	0.26	29.19	0.13	0.99	1.36
Max	3140.57	1678.56	201.23	7239.93	11.93	1.84	167.51	1.70	12.30	185.00
TPV > 30 mL										
Median	590.18	451.35	63.62	641.25	1.56	0.70	79.70	0.79	14.45	10.20
Min	176.46	151.04	11.06	220.09	0.83	0.004	15.61	0.17	4.10	4.90
Max	5814.15	1652.59	186.66	2206.79	6.58	3.33	219.62	3.12	24.95	346.00
*p*-value	n.s.	n.s.	n.s.	n.s.	n.s.	n.s.	n.s.	*	*	n.s.

TPV—Total prostate volume; Min—minimum value; Max—maximum value. n.s.—non significant. * (asterix)—statistically significant at *p* < 0.05 level, according to the Mann–Whitney U test.

## Data Availability

All data supporting the findings of this study are available from the corresponding author upon reasonable request.

## Supplementary Materials

The following supporting information can be downloaded at: https://www.mdpi.com/article/10.3390/ijms27031466/s1.

## Abbreviations

The following abbreviations are used in this manuscript:

BHP	Benign Prostatic Hyperplasia
TZ	Transitional Zone
ISP-OES	Inductively Coupled Plasma Optical Emission Spectrometry
T	Testosterone
DHT	Dihydrotestosterone
LC-MS	Liquid Chromatography Mass Spectrometry
PRL	Prolactin
CTP	Citrate Transport Protein
ASP	Asparate
OAA	Oxaloacetate
mAAT	Mitochondrial Asparate Aminotransferase
PDH	Pyruvate Dehydrogenase
CS	Citrate synthase
PSA	Prostate-Specific Antigen
PCa	Prostate Cancer
TRUS	Transrectal Ultrasound
TPV	Total Prostate Volume
TURP	Transurethral resection of the prostate
EDXRF	Energy-Dispersive X-ray Fluorescence
RRP	Radical retropubic prostatectomy
PZ	Peripheral Zone
CZ	Central Zone
w.w.	Wet Weight
PTFE	Polytetrafluoroethylene
