# Supplementary material for: Is Zinc Accumulation Increased in Hyperplastic Compared to Normal Prostate Tissue
Source: Int J Mol Sci. 2026 Feb 2;27(3):1466. doi: 10.3390/ijms27031466 (PMC12898189; doi:10.3390/ijms27031466)
Supplement: Supplementary file 1 [file ijms-27-01466-s001.zip › ijms-4107046-supplementary.pdf]

**Supplementary material**

**Table S1.** Measured concentration of elements (μg/g), testosterone (T, ng/g), dihydrotestosterone (DHT, ng/g), and prostate-specific antigen (PSA, ng/mL) in the transition zone tissue of small and large prostates.

| No | Ca | K | Mg | Na | Cu | Mn | Zn | T | DHT | PSA | PH |
|---|---|---|---|---|---|---|---|---|---|---|---|
| | | | | | TPV ≤ 30 mL | | | | | | |
| **1** | 589.66 | 315.82 | 22.44 | 363.91 | 0.88 | 0.64 | 44.18 | 0.23 | 1.03 | 10.77 | m |
| **2** | 2249.72 | 580.60 | 64.38 | 1075.02 | 1.86 | 0.69 | 77.61 | 0.13 | 0.99 | 19.10 | b |
| **3** | 605.21 | 670.70 | 74.61 | 712.55 | 1.98 | 0.65 | 113.44 | 0.62 | 3.69 | - | m |
| **4** | 646.10 | 1060.03 | 171.49 | 484.24 | 1.59 | 0.70 | 40.69 | 0.40 | 2.16 | 185.00 | m |
| **5** | 406.77 | 1678.56 | 168.89 | 684.90 | 2.09 | 0.91 | 55.31 | 0.78 | 3.26 | 27.30 | m |
| **6** | 592.62 | 388.18 | 23.99 | 387.96 | 0.43 | 0.26 | 37.30 | 0.33 | 1.48 | 17.20 | m |
| **7** | 621.95 | 268.60 | 23.86 | 317.05 | 0.84 | 1.09 | 44.55 | 0.55 | 2.73 | 16.00 | b |
| **8** | 703.93 | 1128.69 | 132.24 | 2623.20 | 3.84 | 0.88 | 56.05 | 0.43 | 6.00 | 14.70 | b |
| **9** | 1504.71 | 409.70 | 46.61 | 612.81 | 3.21 | 0.72 | 87.77 | 0.19 | 1.44 | 8.58 | m |
| **10** | 408.59 | 460.85 | 57.50 | 855.57 | 1.28 | 1.01 | 48.84 | 0.24 | 2.33 | 24.00 | b |
| **11** | 1542.89 | 582.30 | 59.07 | 741.06 | 4.83 | 0.63 | 75.77 | 0.19 | 2.73 | 20.41 | m |
| **12** | 833.80 | 763.17 | 135.74 | 740.94 | 1.96 | 1.09 | 110.54 | 0.47 | 3.90 | 7.60 | m |
| **13** | 1741.66 | 480.55 | 45.11 | 559.66 | 1.40 | 0.78 | 90.21 | 0.81 | 3.25 | 8.48 | b |
| **14** | 671.52 | 494.51 | 92.66 | 604.20 | 1.63 | 0.84 | 38.90 | 0.50 | 1.64 | 6.93 | b |
| **15** | 334.90 | 220.11 | 15.80 | 239.59 | 2.89 | 0.60 | 101.90 | 0.24 | 1.52 | 6.70 | m |
| **16** | 640.48 | 345.18 | 68.69 | 510.08 | 3.65 | 0.90 | 86.37 | 0.50 | 4.95 | 6.62 | m |
| **17** | 1355.32 | 351.09 | 65.05 | 596.71 | 3.88 | 1.02 | 85.96 | 0.44 | 4.71 | 7.62 | b |
| **18** | 605.62 | 874.92 | 115.34 | 7239.93 | 1.95 | 0.69 | 64.31 | 0.45 | 1.56 | 10.80 | m |
| **19** | 382.85 | 403.57 | 51.18 | 538.09 | 1.57 | 0.48 | 68.78 | 1.70 | 9.18 | 8.18 | b |
| **20** | 462.13 | 515.27 | 75.56 | 570.25 | 1.76 | 0.69 | 39.74 | 0.44 | 2.25 | 1.80 | b |
| **21** | 2771.95 | 1462.45 | 201.23 | 1531.12 | 3.22 | 1.69 | 159.74 | 0.55 | 9.78 | 95.33 | m |
| **22** | 1147.33 | 240.14 | 27.71 | 243.99 | 0.74 | 0.52 | 55.39 | 0.40 | 3.25 | 12.00 | b |
| **23** | 647.00 | 648.29 | 87.56 | 969.81 | 2.27 | 0.89 | 105.51 | 0.63 | 6.40 | 8.79 | b |
| **24** | 565.90 | 437.33 | 28.87 | 458.91 | 2.81 | 0.45 | 94.64 | 0.28 | 4.14 | 8.32 | m |
| **25** | 513.63 | 1537.62 | 120.05 | 1928.54 | 1.78 | 0.62 | 47.45 | 0.84 | 12.30 | 14.62 | m |
| **26** | 326.42 | 623.54 | 85.36 | 1105.41 | 3.66 | 0.98 | 46.49 | 0.44 | 6.80 | 18.00 | b |
| **27** | 553.06 | 794.43 | 155.75 | 879.45 | 3.03 | 0.75 | 45.90 | 0.29 | 9.25 | 2.40 | m |
| **28** | 507.78 | 340.30 | 83.88 | 659.40 | 1.95 | 1.67 | 45.80 | 0.18 | 7.69 | 7.40 | m |
| **29** | 500.02 | 360.17 | 28.51 | 387.25 | 11.93 | 1.27 | 115.49 | 0.29 | 6.34 | 6.06 | b |
| **30** | 156.53 | 229.65 | 13.64 | 221.72 | 1.35 | 0.50 | 29.19 | 0.34 | 4.76 | 18.00 | m |
| **31** | 300.38 | 265.30 | 14.81 | 303.84 | 1.08 | 0.50 | 34.21 | 0.42 | 5.22 | 17.00 | m |
| **32** | 3140.57 | 891.17 | 192.49 | 5173.73 | 3.48 | 1.84 | 167.51 | 0.37 | 8.61 | 10.50 | m |
| **33** | 401.99 | 481.56 | 82.47 | 640.60 | 1.09 | 0.55 | 29.29 | 1.11 | 6.08 | 5.78 | m |
| **34** | 1482.31 | 673.43 | 157.04 | 914.16 | 3.62 | 1.40 | 143.73 | 0.18 | 6.75 | 1.36 | b |
| **35** | 1399.61 | 479.86 | 36.74 | 452.20 | 1.11 | 0.62 | 93.33 | 0.53 | 5.82 | 9.20 | m |
| **36** | 644.68 | 440.84 | 118.96 | 715.99 | 1.55 | 1.22 | 50.61 | 0.44 | 5.29 | 5.26 | b |
| | | | | | TPV > 30 mL | | | | | | |
| **37** | 745.70 | 650.13 | 112.31 | 1466.55 | 2.40 | 1.35 | 162.69 | 0.48 | 5.33 | 22.03 | m |
| **38** | 4742.84 | 513.82 | 63.14 | 556.67 | 1.45 | 1.21 | 219.62 | 0.38 | 6.01 | 7.76 | b |
| **39** | 484.82 | 1652.59 | 140.66 | 1939.49 | 1.26 | 0.44 | 145.94 | 0.65 | 14.40 | 10.12 | b |

| | | | | | | | | | | | |
|---|---|---|---|---|---|---|---|---|---|---|---|
| **40** | 732.49 | 694.33 | 97.11 | 1929.38 | 2.78 | 1.16 | 120.01 | 0.84 | 7.82 | 4.90 | b |
| **41** | 1130.09 | 503.20 | 47.38 | 377.68 | 1.13 | 0.42 | 51.41 | 1.27 | 9.37 | 9.73 | m |
| **42** | 373.79 | 206.28 | 13.97 | 245.38 | 0.83 | 0.60 | 48.23 | 0.51 | 5.02 | 9.19 | b |
| **43** | 296.44 | 293.65 | 17.31 | 222.66 | 6.58 | 0.45 | 100.07 | 0.31 | 4.85 | 15.45 | m |
| **44** | 315.59 | 352.22 | 63.12 | 540.25 | 1.66 | 0.56 | 23.90 | 0.54 | 4.10 | - | b |
| **45** | 2356.79 | 510.34 | 53.81 | 478.81 | 1.10 | 0.69 | 121.19 | 2.20 | 15.01 | 42.40 | b |
| **46** | 427.10 | 436.22 | 47.43 | 523.04 | 1.99 | 0.52 | 68.40 | 0.27 | 5.99 | - | m |
| **47** | 567.63 | 174.38 | 49.75 | 375.84 | 1.57 | 0.83 | 40.51 | 0.40 | 6.39 | 13.60 | b |
| **48** | 370.26 | 251.74 | 19.79 | 251.67 | 1.26 | 0.67 | 39.96 | 0.25 | 5.49 | 77.50 | m |
| **49** | 553.35 | 513.97 | 58.62 | 891.34 | 1.30 | 0.62 | 89.17 | 0.44 | 5.93 | 5.16 | b |
| **50** | 821.98 | 739.90 | 169.48 | 730.02 | 2.78 | 0.98 | 165.18 | 0.37 | 8.37 | 9.69 | b |
| **51** | 1605.38 | 597.80 | 64.96 | 663.68 | 5.16 | 0.76 | 55.27 | 0.85 | 6.91 | 10.30 | m |
| **52** | 1932.09 | 220.32 | 32.67 | 252.34 | 0.94 | 0.71 | 86.51 | 0.57 | 21.47 | 7.05 | b |
| **53** | 728.93 | 667.18 | 118.67 | 647.75 | 1.71 | 0.95 | 96.64 | 1.19 | 8.95 | 15.01 | m |
| **54** | 656.85 | 473.98 | 76.76 | 677.50 | 3.99 | 0.84 | 30.70 | 0.73 | 21.60 | 7.08 | m |
| **55** | 466.20 | 367.64 | 56.16 | 400.98 | 2.43 | 0.67 | 62.48 | 0.36 | 19.13 | 10.20 | m |
| **56** | 3794.65 | 536.29 | 64.07 | 557.76 | 1.52 | 0.70 | 129.83 | 1.09 | 7.83 | 10.00 | b |
| **57** | 544.64 | 817.44 | 87.52 | 1638.40 | 2.14 | 0.68 | 65.36 | 1.73 | 16.11 | 30.34 | m |
| **58** | 620.63 | 518.12 | 103.40 | 821.71 | 3.68 | 1.43 | 165.59 | 1.23 | 14.65 | - | m |
| **59** | 357.87 | 435.44 | 63.16 | 881.13 | 1.51 | 0.72 | 22.08 | 0.81 | 15.98 | 10.27 | b |
| **60** | 438.09 | 606.13 | 113.66 | 794.24 | 1.40 | 0.69 | 68.31 | 0.82 | 18.43 | 6.98 | m |
| **61** | 382.88 | 679.26 | 94.80 | 597.17 | 1.62 | 0.51 | 27.62 | 1.36 | 20.66 | 87.57 | m |
| **62** | 579.50 | 291.25 | 46.39 | 338.80 | 2.78 | 0.56 | 73.23 | 0.79 | 18.00 | 9.38 | b |
| **63** | 338.88 | 497.82 | 70.80 | 566.53 | 1.88 | 0.46 | 17.71 | 0.66 | 21.32 | 67.84 | m |
| **64** | 665.80 | 1532.57 | 144.84 | 2206.79 | 5.58 | 1.12 | 92.78 | 0.81 | 21.96 | 13.20 | m |
| **65** | 290.29 | 243.66 | 20.84 | 294.43 | 2.64 | 33.30 | 62.42 | 1.70 | 19.40 | 5.10 | b |
| **66** | 681.07 | 678.20 | 106.71 | 724.73 | 1.36 | 0.57 | 122.65 | 0.98 | 15.19 | 11.32 | b |
| **67** | 606.34 | 290.59 | 47.54 | 551.21 | 1.60 | 1.37 | 57.87 | 1.36 | 12.25 | 18.20 | m |
| **68** | 176.46 | 151.04 | 11.06 | 220.09 | 1.09 | 0.68 | 15.61 | 0.72 | 9.30 | 7.32 | b |
| **69** | 600.85 | 569.58 | 92.90 | 777.80 | 1.63 | 0.87 | 68.61 | 1.55 | 14.45 | 8.70 | b |
| **70** | 1621.17 | 465.14 | 77.56 | 1229.35 | 1.37 | 0.69 | 44.82 | 2.22 | 19.35 | 10.19 | b |
| **71** | 2117.91 | 658.56 | 141.67 | 1406.35 | 2.45 | 1.18 | 122.08 | 0.40 | 17.06 | 14.00 | m |
| **72** | 1257.47 | 652.14 | 126.44 | 987.66 | 1.95 | 2.14 | 103.14 | 2.11 | 20.09 | 5.91 | b |
| **73** | 1281.02 | 556.24 | 97.48 | 1046.18 | 1.59 | 1.56 | 59.23 | 2.05 | 18.85 | 6.50 | m |
| **74** | 307.29 | 355.35 | 44.61 | 472.40 | 1.55 | 0.56 | 61.75 | 2.27 | 18.97 | 13.00 | b |
| **75** | 5814.15 | 685.36 | 186.66 | 784.95 | 1.80 | 1.43 | 160.46 | 0.63 | 20.33 | 150.00 | m |
| **76** | 399.66 | 344.46 | 22.89 | 298.12 | 1.12 | 0.75 | 53.86 | 0.42 | 21.89 | 346.00 | m |
| **77** | 571.43 | 437.55 | 81.07 | 543.94 | 2.45 | 0.74 | 94.37 | 1.28 | 19.11 | 7.10 | b |
| **78** | 2931.81 | 452.92 | 73.12 | 693.64 | 3.43 | 0.55 | 173.69 | 0.94 | 17.51 | 266.00 | m |
| **79** | 329.16 | 267.35 | 17.08 | 257.61 | 2.19 | 0.51 | 51.02 | 1.47 | 21.35 | 9.35 | b |
| **80** | 624.35 | 595.47 | 93.02 | 954.66 | 1.79 | 0.83 | 98.99 | 0.17 | 19.53 | 7.97 | b |
| **81** | 1691.58 | 368.99 | 72.37 | 1885.01 | 1.29 | 0.91 | 86.17 | 2.87 | 24.95 | 20.83 | b |
| **82** | 1188.66 | 498.48 | 41.91 | 425.55 | 1.00 | 0.51 | 120.47 | 3.12 | 20.60 | 15.62 | m |

TPV - Total prostate volume; TPV ≤ 30 mL - ransition zone tissue of small prostates; TPV > 31 mL - transition zone tissue of large prostates; PH —pathohistology (b - benign; m - malignant); „-“ - not determined.

1. **Correlation analysis**
    1.1. **Correlation between serum prostate-specific antigen (PSA) values and elemental concentrations**

Correlation analyses were performed between serum prostate-specific antigen (PSA) values and prostate tissue zinc concentrations, as well as with other analyzed elements (Figure S1). The correlation between PSA values and Zn concentrations was not proven; namely, in the entire group there were also patients with very high PSA levels and proven metastases. However, this correlation was not proven neither in the group of patients with benign histology in the peripheral zone.

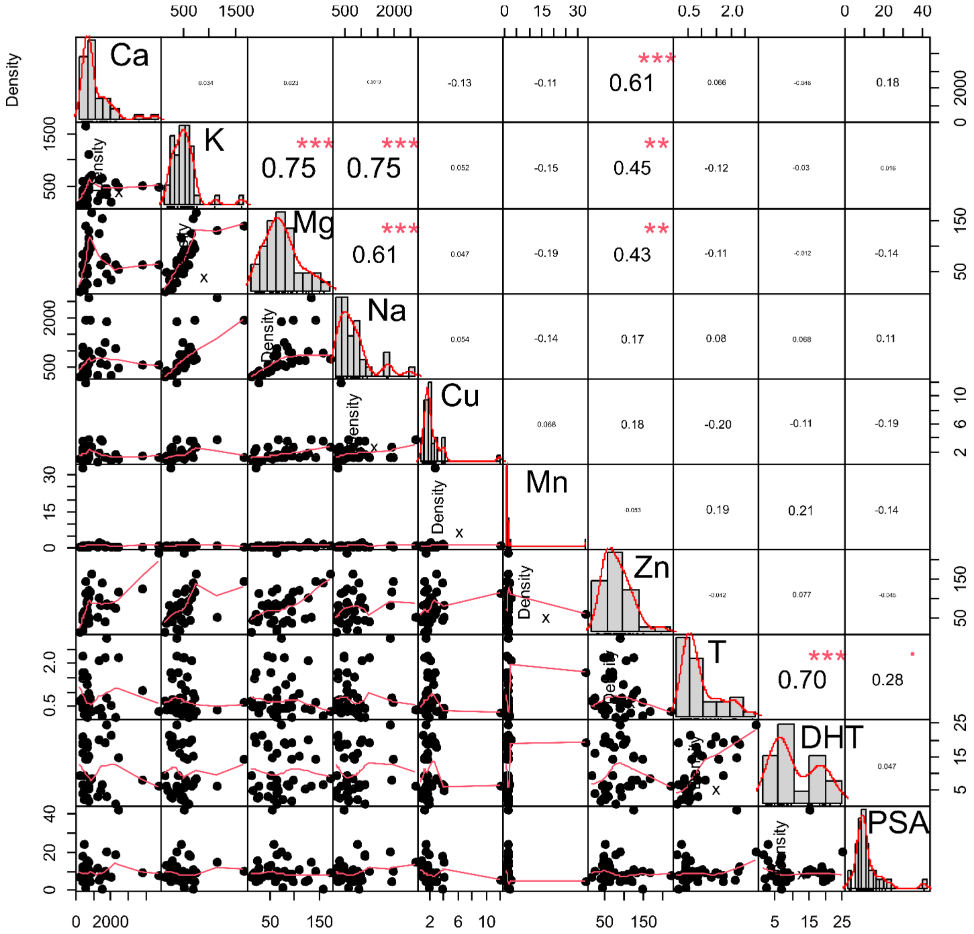


**Figure S1.** Correlation matrix of elemental and hormonal concentrations in prostatic transition-zone tissue.

The correlation analysis revealed several statistically significant associations among elemental concentrations and hormonal parameters (Figure S1).

A strong positive correlation was observed between potassium (K) and magnesium (Mg) ($r = 0.754$, $p < 0.001$), as well as between K and sodium (Na) ($r = 0.747$, $p < 0.001$), indicating a close interrelationship among these macroelements. Potassium also showed a moderate positive correlation with zinc (Zn) ($r = 0.441$, $p = 0.006$).

Magnesium was moderately and positively correlated with sodium (r = 0.613, p < 0.001) and zinc (r = 0.437, p = 0.006), suggesting coordinated variation of these elements within the analysed samples.
Calcium (Ca) exhibited a moderate positive correlation with zinc (r = 0.600, p < 0.001), indicating that higher calcium levels were associated with higher zinc concentrations.
Regarding hormonal parameters, testosterone (T) showed a strong positive correlation with dihydrotestosterone (DHT) (r = 0.698, p < 0.001), reflecting their expected biological relationship.

### 1.2. Correlation between elemental and hormonal concentration in prostatic transition-zone tissue across volume categories (≤ 20 mL, 20–40 mL, > 40 mL)

An additional analysis was performed using an alternative grading system based on TPV categories (≤ 20 g, 20–40 g, and > 40 g) (Table S1). The results of this analysis are presented as correlation matrices for each TPV group. The applied stratification revealed clearer and more consistent associations involving zinc, particularly in the moderate and high TPV groups, suggesting that finer TPV categorization may indeed improve the detection of underlying relationships. However, despite these improvements, the observed trends did not uniformly translate into statistically significant differences in zinc concentration between TPV groups, indicating that biological variability and sample size remain important limiting factors.

**Table S2.** The descriptive statistical analysis of seven elements (µg/g), testosterone (T, ng/g), dihydrotestosterone (DHT, ng/g), and prostate-specific antigen (PSA, ng/mL), measured in the transition zone tissue across volume categories (≤ 20 mL, 20–40 mL, > 40 mL).

| Parameter | Ca | K | Mg | Na | Cu | Mn | Zn | T | DHT | PSA |
|---|---|---|---|---|---|---|---|---|---|---|
| | | | | | TPV ≤ 20 g | | | | | |
| **Median** | 634.02 | 581.45 | 61.72 | 698.73 | 1.91 | 0.71 | 55.68 | 0.37 | 2.53 | 17.20 |
| **Min** | 406.77 | 268.60 | 22.44 | 317.05 | 0.43 | 0.26 | 37.30 | 0.13 | 0.99 | 7.60 |
| **Max** | 2249.72 | 1678.56 | 171.49 | 2623.20 | 4.83 | 1.09 | 113.44 | 0.78 | 6.00 | 185.00 |
| | | | | | TPV in range 20–40 g | | | | | |
| **Median** | 605.62 | 481.56 | 64.96 | 570.25 | 1.76 | 0.71 | 85.96 | 0.44 | 6.01 | 9.19 |
| **Min** | 156.53 | 174.38 | 13.64 | 221.72 | 0.74 | 0.42 | 23.90 | 0.18 | 1.52 | 1.36 |
| **Max** | 4742.84 | 1652.59 | 201.23 | 7239.93 | 11.93 | 1.84 | 219.62 | 2.20 | 21.47 | 95.33 |
| | | | | | TPV > 40 g | | | | | |
| **Median** | 606.34 | 497.82 | 76.76 | 693.64 | 1.79 | 0.72 | 68.31 | 1.09 | 19.11 | 10.24 |
| **Min** | 176.46 | 151.04 | 11.06 | 220.09 | 1.00 | 0.46 | 15.61 | 0.17 | 7.83 | 5.10 |
| **Max** | 5814.15 | 1532.57 | 186.66 | 2206.79 | 5.58 | 3.33 | 173.69 | 3.12 | 24.95 | 346.00 |

TPV - Total prostate volume; Min - minimum value; Max - maximum value. n.s. – non significant. * (asterix) - statistically significant at $p < 0.05$ level, according to the Mann–Whitney U test.

The detailed correlation analysis provided a comprehensive numerical overview of these inter-element relationships (Figure S2, Figure S3 and Figure S4, for TPV ≤ 20 g, TPV in range 20–40 g, and TPV > 40 g, respectively).

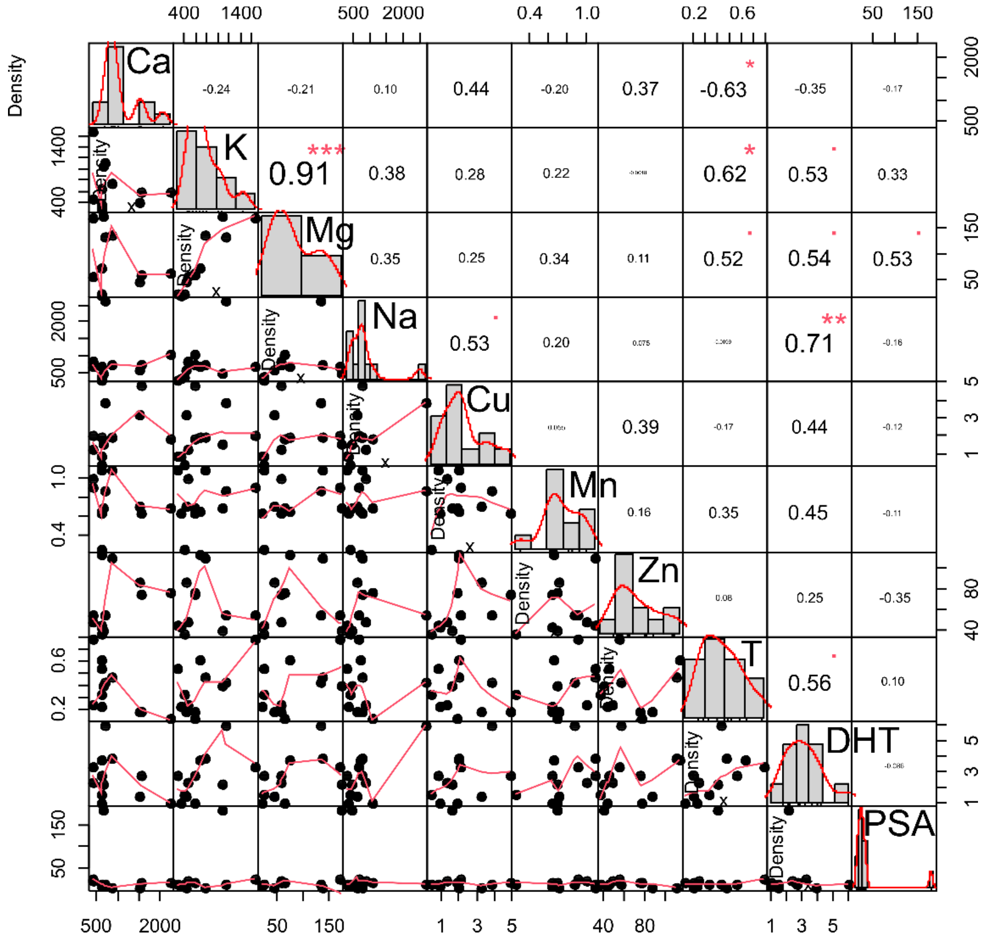

**Figure S2.** Correlation matrix of elemental and hormonal concentrations in prostatic transition-zone tissue (n = 12), for TPV ≤ 20 g.

For samples with TPV ≤ 20 g, several statistically significant correlations were observed among mineral elements and DHT. Calcium was moderately and negatively correlated with magnesium ($r = -0.616$, $p = 0.043$), indicating an inverse association between these two elements at lower TPV levels. Potassium showed strong positive correlations with magnesium ($r = 0.908$, $p < 0.001$) and temperature ($r = 0.671$, $p = 0.024$), suggesting coordinated variation of these parameters. In addition, sodium exhibited a significant positive correlation with DHT ($r = 0.741$, $p = 0.009$), implying a potential link between sodium content and thermal-related changes in this TPV range.

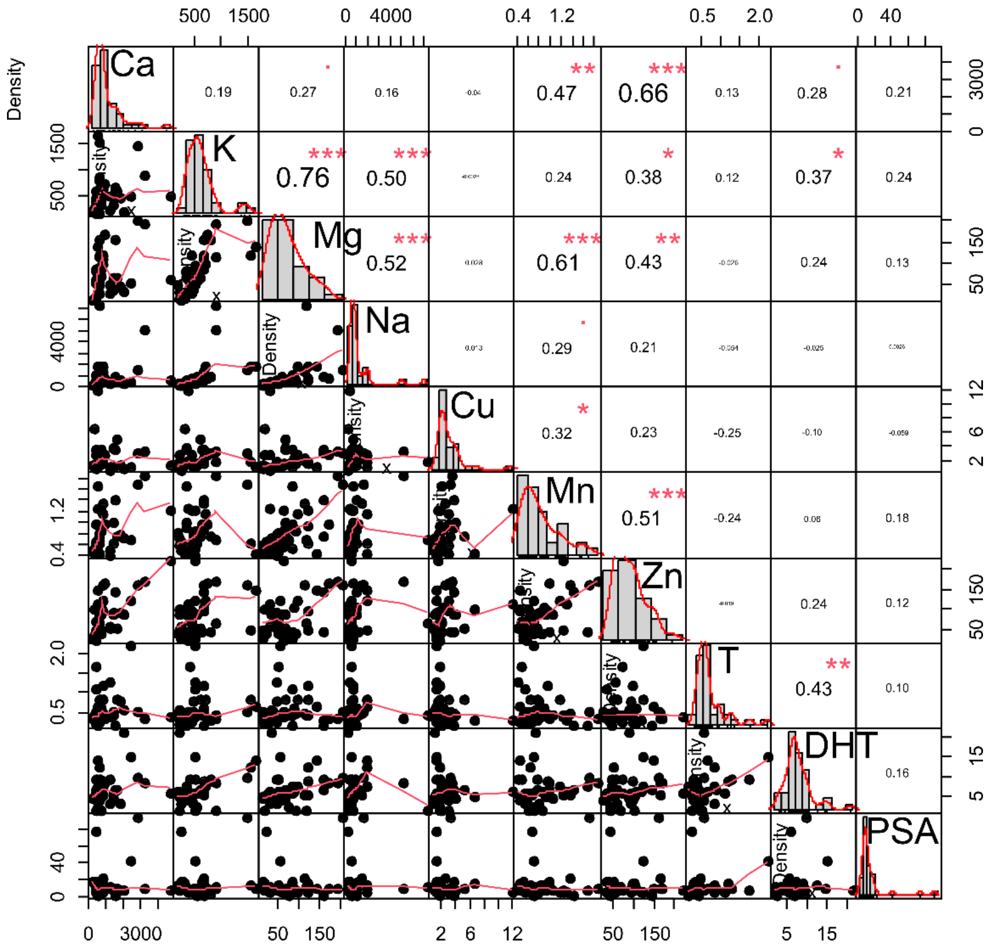


**Figure S3.** Correlation matrix of elemental and hormonal concentrations in prostatic transition-zone tissue (n = 41), for TPV in range 20–40 g.

In the intermediate TPV range (20–40 g), a more complex network of significant positive correlations was detected. Calcium was positively associated with zinc (r = 0.456, p = 0.003) and DHT (r = 0.651, p < 0.001). Potassium exhibited strong correlations with magnesium (r = 0.757, p < 0.001) and sodium (r = 0.497, p = 0.001), as well as weaker but statistically significant associations with zinc (r = 0.368, p = 0.021) and DHT (r = 0.361, p = 0.024). Magnesium showed significant positive correlations with sodium (r = 0.521, p = 0.001), manganese (r = 0.607, p < 0.001), and zinc (r = 0.427, p = 0.007). Furthermore, manganese was positively correlated with zinc (r = 0.501, p = 0.001), while temperature was also positively associated with zinc (r = 0.427, p = 0.007). These results indicate a pronounced interdependence among mineral elements and thermal parameters within the moderate TPV range.

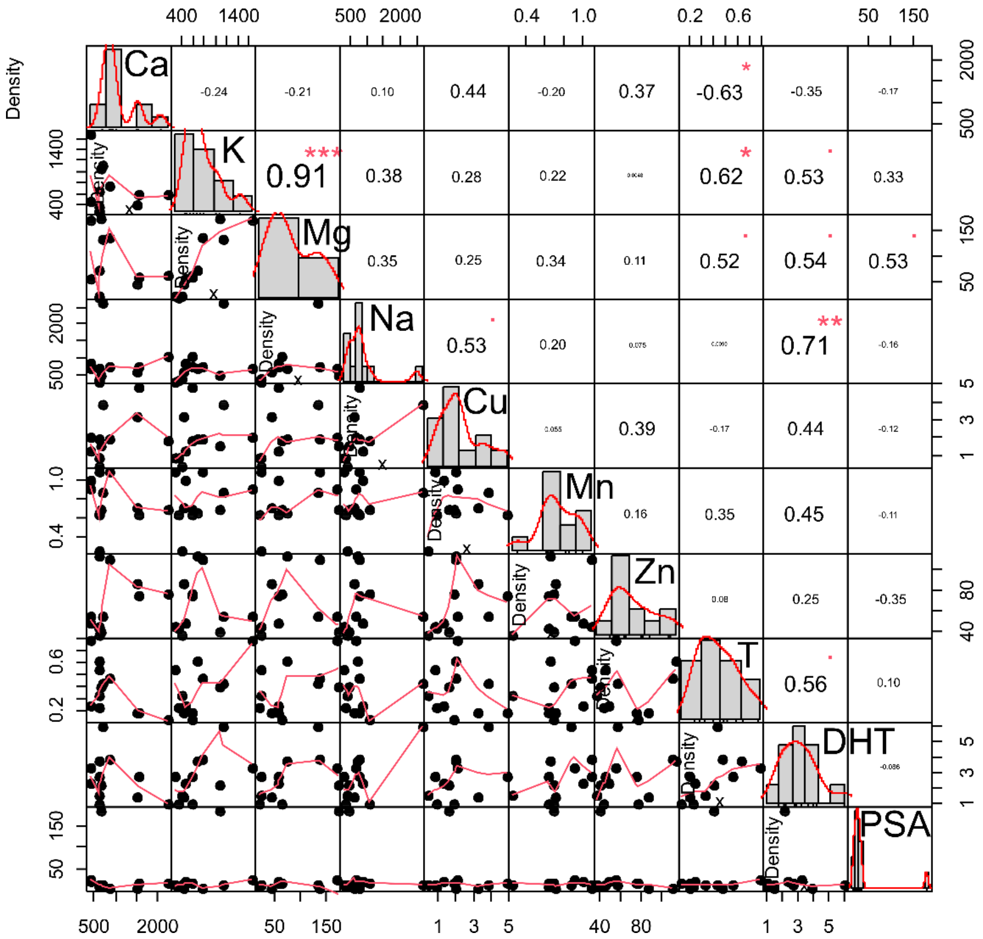

**Figure S4.** Correlation matrix of elemental and hormonal concentrations in prostatic transition-zone tissue (n = 29), for TPV > 40 g.

At TPV values greater than 40 g, strong and statistically significant positive correlations persisted among several mineral elements. Calcium was positively correlated with magnesium (r = 0.516, p = 0.005) and zinc (r = 0.720, p < 0.001). Potassium exhibited strong associations with magnesium (r = 0.734, p < 0.001), sodium (r = 0.722, p < 0.001), and copper (r = 0.530, p = 0.004). Magnesium was also positively correlated with sodium (r = 0.600, p = 0.001) and zinc (r = 0.482, p = 0.009).

2. **ICP-OES measurements**

**2.1. Wavelengths used for quantification.**

The emission lines employed in the ICP-OES analysis were selected based on an internal interference assessment conducted during method development. For each analyte, two to three emission lines were monitored simultaneously across all samples. The final selection of wavelengths for quantification was made by comparing sample and standard spectra, taking into account the absence of spectral overlaps, signal intensity, signal-to-background ratio, and the quality of the calibration curve fit. Furthermore, the

full width at half maximum (FWHM) of each emission line was considered to ensure adequate spectral resolution and to minimize potential inter-element interferences. The instrumental operating conditions for ICP-OES are listed in Table S3. The exact wavelengths used are listed in Table S4.

**Table S3**. Instrumental operating conditions for ICP-OES.

| *Parameter* | **Value** |
|---|---|
| Radio frequency power (RF) | 1350 W |
| Plasma view | Axial |
| Nebulizer | Standard alumina concentric |
| Spray chamber | Standard glass cyclonic |
| Pump tubing (Tygon®) | Sample (Orange-White)<br>Drain (White-White) |
| Ceramic centre tube | 2 mm |
| Purge gas | Argon |
| Nebulizer argon flow | 0.50 L/min |
| Auxiliary argon flow | 0.5 L/min |
| Coolant argon flow | 12 L/min |
| Sample flush time | 30 s |
| Analysis pump rate | 50 rpm |
| Integration times | |
| Low (166 -230 nm) | 15 s |
| High (230-847 nm) | 5 s |
| Analysis mode | Speed |
| Number of repetitions | n=3 |
| Calibration type | External |
| Fit type | Linear |
| Weighting | 1/Conc |
| Software | iTEVA |
| Emission Wavelengths | given in Table S4 |

### 2.2. Limits of Detection and Quantification.

The limits of detection (LOD) and quantification (LOQ) were automatically calculated by the iTEVA software (Thermo Scientific) based on calibration curve statistics (3σ and 10σ criteria, respectively), and ranged from 0.022 to 0.932 μg/L for LOD and from 0.183 to 7.767 μg/L for LOQ. Individual LOD and LOQ values for each element are listed in Table S4, expressed both in μg/L solution and in μg/g of the prostate tissue, calculated based on the sample preparation protocol (25 mg in 10 mL).

**Table S4-a**. Parameters: calibration curves for ICP-OES analysis (Fit type: Linear; Weighting: 1/Conc)

| **Element –** emission line* | **Range** μg/L | **Correlation coefficients ($R^2$)** | **RSD (%)** | **Slope / Sensitivity** cps·L/μg** | **LOD (μg/L)** | **LOQ (μg/L)** |
|---|---|---|---|---|---|---|
| Zn II 202.548 nm | 1-1000 | 0.99860 | <0.5 | 29.459 | 0.055 | 0.183 |
| Ca II 315.887 nm | 10-10000 | 0.99947 | <0.5 | 6.010 | 2.116 | 7.053 |
| Cu II 224.700 nm | 1-1000 | 0.99999 | <0.5 | 5.601 | 0.501 | 1.671 |
| K I 769.896 nm | 10-10000 | 0.99935 | <0.5 | 18.399 | 2.330 | 7.767 |
| Mg I 285.213 nm | 1-10000 | 0.99927 | <0.5 | 19.564 | 0.438 | 1.461 |
| Mn II 257.610 nm | 1-1000 | 0.99975 | <0.5 | 24.208 | 0.212 | 0.707 |
| Na I 589.592 nm | 10-10000 | 0.99684 | <0.5 | 117.985 | 0.421 | 1.404 |

* Spectroscopic marking of the emission line:

I: atomic line - thermal state I

II: ionic line - thermal state II

** Signal intensity (y-axis) expressed as counts per second (cps)

**Table S4-b**. Limits of detection (LOD) and quantification (LOQ) for ICP-OES analysis. Values are expressed in μg/L (solution) and recalculated to μg/g w.w. prostate tissue (based on the 25 mg sample diluted to a final volume of 10 mL).

| **Element -** emission line* | **LOD (μg/L)** | **LOQ (μg/L)** | **LOD (μg/g)** | **LOQ (μg/g)** |
|---|---|---|---|---|
| Zn II 202.548 nm | 0.055 | 0.183 | 0.022 | 0.073 |
| Ca II 315.887 nm | 2.116 | 7.053 | 0.846 | 2.821 |
| Cu II 224.700 nm | 0.501 | 1.671 | 0.200 | 0.668 |
| K I 769.896 nm | 2.330 | 7.767 | 0.932 | 3.107 |
| Mg I 285.213 nm | 0.438 | 1.461 | 0.175 | 0.584 |
| Mn II 257.610 nm | 0.212 | 0.707 | 0.085 | 0.283 |
| Na I 589.592 nm | 0.421 | 1.404 | 0.168 | 0.562 |

### 2.3. Accuracy

Accuracy of the ICP-OES measurements was assessed using a certified reference material for trace metals in fish protein (DORM-4, National Research Council Canada, Ottawa, ON, Canada), derived from dolphin muscle tissue. The agreement between measured and certified values confirmed the accuracy of the method, with recoveries ranging from 97% to 102%.

### 2.4. Precision

Method precision was evaluated through replicate analyses. The relative standard deviations of the mean values obtained from triplicate measurements were lower than 0.5%, indicating excellent repeatability.

### 2.5. Sensitivity

Sensitivity was defined as the slope of the calibration curve obtained for each element and depended on the selected emission line. The calculated slope values ranged from 5.601 to 117.985 cps·L·$\mu g^{-1}$, demonstrating adequate sensitivity of the method for trace-level elemental determination.

### 2.6. Quality Control (QC)

Quality control procedures included the analysis of blank samples, the use of matrix-matched calibration solutions, and triplicate measurements of each sample. Calibration curves were linear over the investigated concentration ranges, with correlation coefficients ($R^2$) exceeding 0.99 for all analyzed elements, confirming the robustness and reliability of the analytical method.

### 2.7. Certified Reference Material (CRM)

**Table S5**. Certified and Measured Concentrations of the Analyzed Elements in the Certified Reference Material for Trace Metals in Fish Protein (DORM-4, National Research Council Canada, Ottawa, ON, Canada; Dolphin Muscle Tissue), (µg/g w.w.)

| Trace metal | Certified Concentrations (µg/g) | Measured Concentrations (µg/g) | Recovery (%) |
|---|---|---|---|
| Cupper (Cu) | 15.9 ± 0.9 | 15.7 ± 0.5 | **98.7** |
| Zink (Zn) | 52.2 ± 3.2 | 51.9 ± 2.2 | **99.4** |
